# Endocrine and Growth Disorders in Taiwanese Children With 22q11.2 Deletion Syndrome

**DOI:** 10.3389/fendo.2022.771100

**Published:** 2022-03-31

**Authors:** Han-Yi Lin, Wen-Yu Tsai, Yi-Ching Tung, Shih-Yao Liu, Ni-Chung Lee, Yin-Hsiu Chien, Wuh-Liang Hwu, Cheng-Ting Lee

**Affiliations:** ^1^ Department of Pediatrics, National Taiwan University Hospital and National Taiwan University College of Medicine, Taipei, Taiwan; ^2^ Department of Medical Genetics, National Taiwan University Hospital, Taipei, Taiwan; ^3^ Graduate Institute of Clinical Medicine, National Taiwan University College of Medicine, Taipei, Taiwan

**Keywords:** 22q11.2 deletion syndrome, DiGeorge syndrome, hypoparathyroidism, thyroid disorders, growth disorders

## Abstract

**Background:**

Endocrine disorders are common in patients with 22q11.2 deletion syndrome (22q11.2DS). This study aimed to elucidate the clinical manifestations of endocrine disorders, including parathyroid, thyroid and growth disorders, in Taiwanese patients with 22q11.2DS.

**Methods:**

From 1994 to 2020, the medical records of 138 patients with 22q11.2DS diagnosed at a tertiary referral medical center in Taiwan were thoroughly reviewed retrospectively.

**Results:**

Hypocalcemia was detected in 57 of 135 patients (42%); 33 of 104 patients (32%) had hypoparathyroidism, and in 87% of them, hypocalcemia was detected before the age of one. Most patients had precipitating stressors during symptomatic hypocalcemic episodes. Eighteen of 29 patients had overt hypoparathyroidism at the last visit: 11 had persistent hypoparathyroidism and the other seven had recurrent hypoparathyroidism. Four of 84 patients had thyroid disorders, including thyroid developmental anomalies in two, dyshormonogenesis in one and Graves’ disease in one. Fifty of 126 patients (40%) had short stature. Age (odds ratio (OR) 0.91; 95% confidence interval (CI) 0.86-0.96; *P*<0.001) and airway anomalies (OR 2.75; 95% CI 1.04-7.31; *P*<0.05) were significant risk factors for short stature in multivariate logistic regression model. Twenty-eight of the 30 patients with airway anomalies were associated with severe congenital heart disease. Adult height standard deviation score (SDS) in 19 patients was significantly lower than target height SDS (-1.15 ± 0.90 vs -0.08 ± 0.65, *P*<0.001).

**Conclusions:**

Hypoparathyroidism is a common endocrine disorder in patients with 22q11.2DS. It is prudent to assess parathyroid function at diagnosis and during follow-up, especially in the presence of stress, to prevent symptomatic hypocalcemia. Although thyroid disorders are not so common as hypoparathyroidism, screening of thyroid dysfunction is justified in these patients. Patients with 22q11.2DS demonstrate a retarded growth pattern with a tendency of catch-up and regular monitoring of growth is indicated.

## Introduction

22q11.2 deletion syndrome (22q11.2 DS) is the most common chromosomal microdeletion disorder, causing multiorgan malformations, including facial dysmorphism, cardiac and palatal abnormalities, immune dysfunction, endocrine, genitourinary and gastrointestinal problems, developmental delay and neuropsychiatric illness (1, 2). Patients with chromosome 22q11.2 deletion may be diagnosed with DiGeorge syndrome, velocardiofacial syndrome, conotruncal anomaly face syndrome, Opitz GBBB syndrome or Cayler cardiofacial syndrome according to phenotype. In terms of endocrine disorders, individuals with 22q11.2DS may present with hypocalcemia due to hypoplasia of the parathyroid glands, thyroid disorders and short stature (3).

Hypocalcemia is the most common manifestation of endocrine disorders, with a prevalence estimated at 32%-80% (1, 4–6). Clinical manifestations and the natural course of hypoparathyroidism vary widely. Thyroid disorders are caused by either autoimmune processes or congenital malformations of thyroid lobes (3). Growth failure correlates with congenital heart defects (7) or feeding problems (1, 8), and the prevalence of growth hormone deficiency has been reported to be 4% (9). Previous studies have found that patients with 22q11.2DS experience retarded growth during childhood followed by a period of catch-up growth (8, 10). Nevertheless, the final adult height is still in the low-normal range of normal adults.

This retrospective case note analysis was conducted to elucidate the clinical manifestations and natural course of endocrine disorders in patients with 22q11.2DS.

## Patients and Methods

This retrospective study was approved by the Institutional Review Board of National Taiwan University Hospital. From 1994 to 2020, 156 patients younger than 18 years were confirmed to have 22q11.2DS at the Department of Pediatrics of National Taiwan University Hospital. Among them, 138 patients (86 males and 52 females) who had detailed medical records for analysis were enrolled in this study. The mean age at diagnosis was 2.3 ± 3.2 years old (range from one day to 17 years and 11 months). The diagnosis of 22q11.2DS was based on chromosomes in 20 patients, fluorescent *in situ* hybridization (FISH) with the TUPLE1 probe in 49, multiplex ligation-dependent probe amplification (MLPA) by SALSA^®^MLPA^®^Probemix P250 DiGeorge (MRC-Holland, Amsterdam, The Netherlands) in 44 and array comparative genomic hybridization (aCGH) in 25. The medical records of these 138 patients, including data for height (Ht), weight (Wt), serum total calcium, phosphorus, magnesium, intact parathyroid hormone (iPTH), alkaline phosphatase (ALP), creatinine, free T4 (FT4), thyroid-stimulating hormone (TSH), growth hormone (GH) and insulin-like growth factor-1 (IGF-1), were thoroughly reviewed.

Serum total calcium, phosphorus, magnesium, creatinine and ALP were assayed using an automatic biochemical analyzer (Beckman Coulter, Indianapolis, IN, USA). Serum iPTH levels were measured by chemiluminescence assay (ARCHITECT system, Abbott, North Chicago, IL, USA). FT4 and TSH concentrations were assessed using chemiluminescent microparticle immunoassay (ARCHITECT system, Abbott, North Chicago, IL, USA). GH and IGF-1 levels were assessed by chemiluminescent immunometric assay (IMMULITE^®^2000 Systems Analyzers, Siemens, Erlangen, Germany). An adequate GH response was defined as a peak GH level over 7 ng/mL after the stimulation test.

The iPTH assay was available at our hospital in 1995, which enables evaluation of the parathyroid status of these patients. For the purpose of this study, only the first data of iPTH levels measured concomitantly with serum total calcium levels were included for analysis. At our hospital, the pediatric normal range for serum total calcium levels was 2.15–2.60 mmol/L, and the pediatric normal range for iPTH levels was 12–72 pg/mL. We considered hypoparathyroidism to be present if iPTH level was below the normal range or was within the normal range in the presence of hypocalcemia after the exclusion of the conditions that may influence parathyroid function such as maternal hyperparathyroidism, infant of diabetic mother or hypomagnesemia etc.

Height and weight in patients with 22q11.2DS were compared with the growth chart of Taiwanese children (11). Short stature was defined as greater than two standard deviations (SDs) below the mean height according to sex and chronological age. Body mass index (BMI) was calculated as weight in kilograms divided by square of height in meters. Small for gestational age (SGA) was defined as birth weight greater than 2 SDs below the mean weight according to the reference of Taiwanese neonates (12). Target height was calculated according to Tanner’s criteria (13), and adult height was defined as growth velocity less than 1 cm in the previous year before the last visit or chronological age older than 20 (14).

Screening of major systemic problem and anthropometric measurement in patients with 22q11.2 DS was done at diagnosis and followed up once every 6 months in this institution. In those with hypoparathyroidism and thyroid dysfunction, blood tests have been followed up once every three months at pediatric endocrine clinic.

### Statistics

Data for continuous variables are expressed as the mean ± SD. Wilcoxon rank sum test was used to compare ordinal data and Chi-square was used to compare categorical data. Independent sample *t*-tests and analysis of variance (ANOVA) tests were employed to evaluate differences in continuous measurements between various categorical variables. Logistic regression was applied to evaluate the risk factors for short stature. We applied the Pearson correlation coefficient to evaluate correlations between continuous variables. Generalized estimating equation was used to model longitudinal data. Statistical analysis was performed using SAS 9.4 for Windows (SAS Institute Inc, Cary, NC, USA). A *P*-value <0.05 was considered significant.

## Results

### Hypocalcemia

Serum total calcium was evaluated in 135 patients in this study, 57 of whom (42%) had hypocalcemia. Twenty-seven of these 57 patients were suspected to have hypocalcemia related to underlying systemic diseases, that may interfere with calcium metabolism, including heart failure with poor feeding in nine, hypomagnesemia in five, respiratory distress in four, acute renal failure in four, blue spells in two, post operation in two and sepsis in one. These patients became normocalcemic with serum iPTH levels in the upper half of normal range after the underlying causes were resolved during follow-up. In the other 30 patients, hypocalcemia was considered to be due to hypoparathyroidism per se after the exclusion of conditions that may interfere with calcium metabolism. Neonatal hypocalcemia, defined as diagnosed within the first month of age, was noted in 18 of 30 patients (60%); eight patients (27%) had hypocalcemia occurring between one and twelve months of age and two between the age of one and ten years, whereas hypocalcemia was detected at an age older than ten years in other two patients.

**Figure 1:**
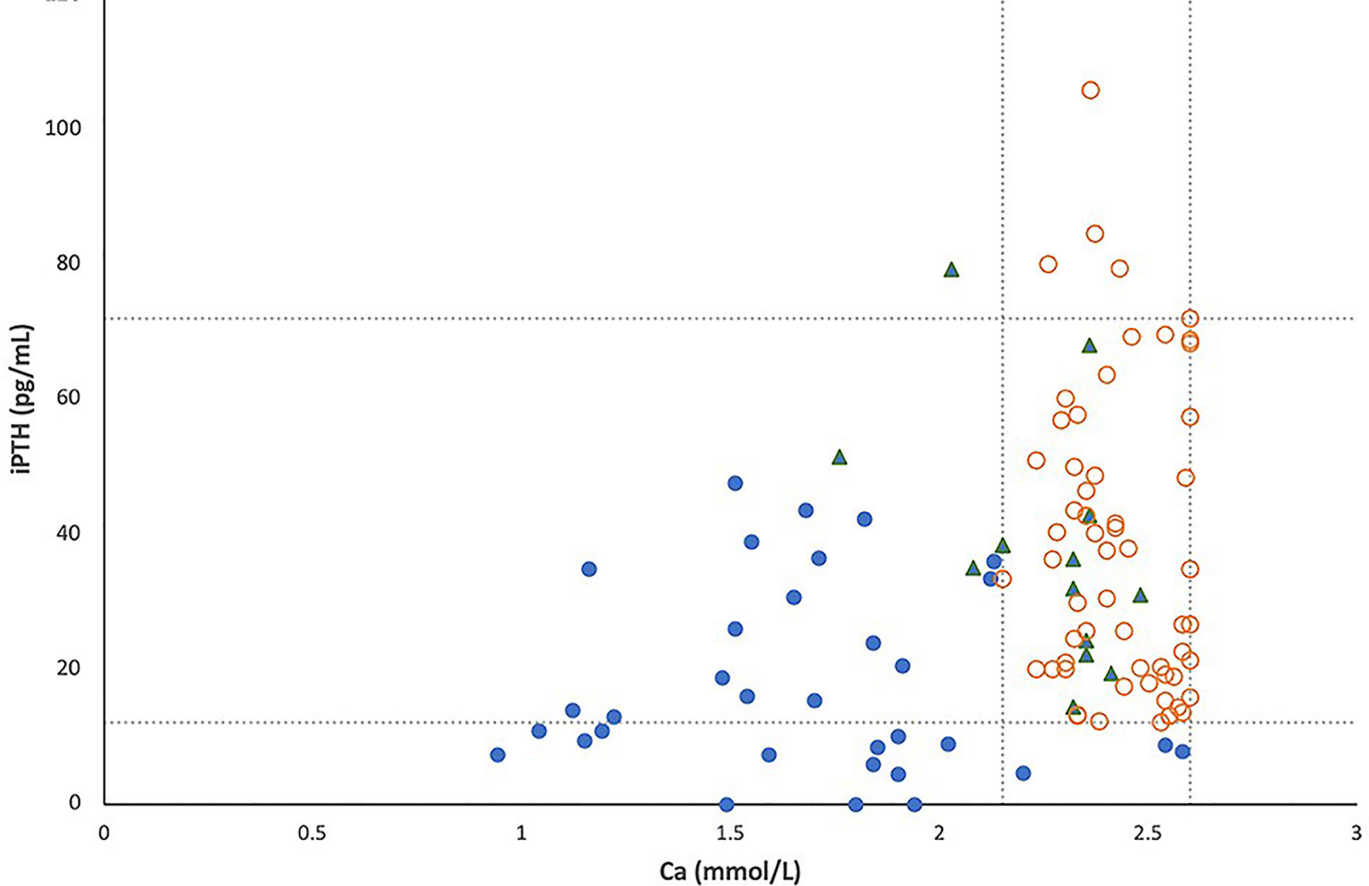
The relationship between serum total calcium and intact PTH levels in 104 patients with 22q11.2 deletion syndrome, including 33 patients with hypoparathyroidism (●), 13 patients with a history of hypocalcemia due to other causes (▲) and 58 patients with normal parathyroid reserve (○). Lines represent pediatric normal ranges of total calcium and intact PTH levels.

All simultaneous serum iPTH and calcium levels obtained for the 104 patients with 22q11.2DS are shown in **Figure 1**, in which lines represent pediatric normal ranges for serum calcium and iPTH levels at our hospital. Among these 104 patients, hypocalcemia was detected in 33 (32%) and low iPTH levels in 16 (15%). In total, 33 patients (22 males, 11 females) were considered to have hypoparathyroidism (32%); 30 of these patients exhibited serum calcium levels below the normal range, as occurred for iPTH levels in 16 of the 33 patients (48%).

Eighteen of the 33 patients (55%) with hypoparathyroidism had symptomatic hypocalcemia at diagnosis. Initial presentations included seizures in 16 patients, carpopedal spasms in one patient and jitteriness in one patient. Their hypocalcemia with low serum iPTH levels persisted after the condition that may interfere with calcium metabolism were corrected. Thirteen of them had a low serum calcium level when iPTH level was assessed during attack. The data are shown in **Table 1**. Six of these 13 patients had low iPTH levels (<12 pg/mL) at the time of hypocalcemia, but none of them had an iPTH level above 39 pg/mL at the time of a hypocalcemic event. Additionally, six of these 13 patients had heart failure, and two were postoperative; three cases were associated with illness, and one patient had hypocalcemia after vigorous exercise. Thus, 12 of 13 (92%) symptomatic hypocalcemic events involved precipitating stressors.

**Table 1:** Simultaneous serum iPTH and calcium levels in 13 patients with symptomatic hypocalcemia.

Patient number	Sex	Age at onset	Ca mmol/L	iPTH pg/mL	Associated clinical status
1	Male	12 days	1.22	13.0	Post colostomy
2	Male	21 days	1.59	7.3	Post colostomy
3	Male	23 days	1.16	34.9	Heart failure
4	Male	30 days	1.19	14.7	Heart failure
5	Female	30 days	1.49	<3.0	Post chemotherapy (Busulfan)
6	Male	13 years	1.84	5.8	
7	Female	13 days	1.04	10.9	Pneumonia
8	Male	7 days	1.12	14.0	Heart failure
9	Female	12 days	1.15	9.4	Heart failure
10	Female	21 days	0.94	7.3	Respiratory distress
11	Male	15 years	1.71	36.4	Post-exercise
12	Female	9 days	1.55	38.9	Heart failure
13	Male	9 days	1.65	30.7	Heart failure

Ca, calcium; iPTH, intact parathyroid hormone.

**Figure 2:**
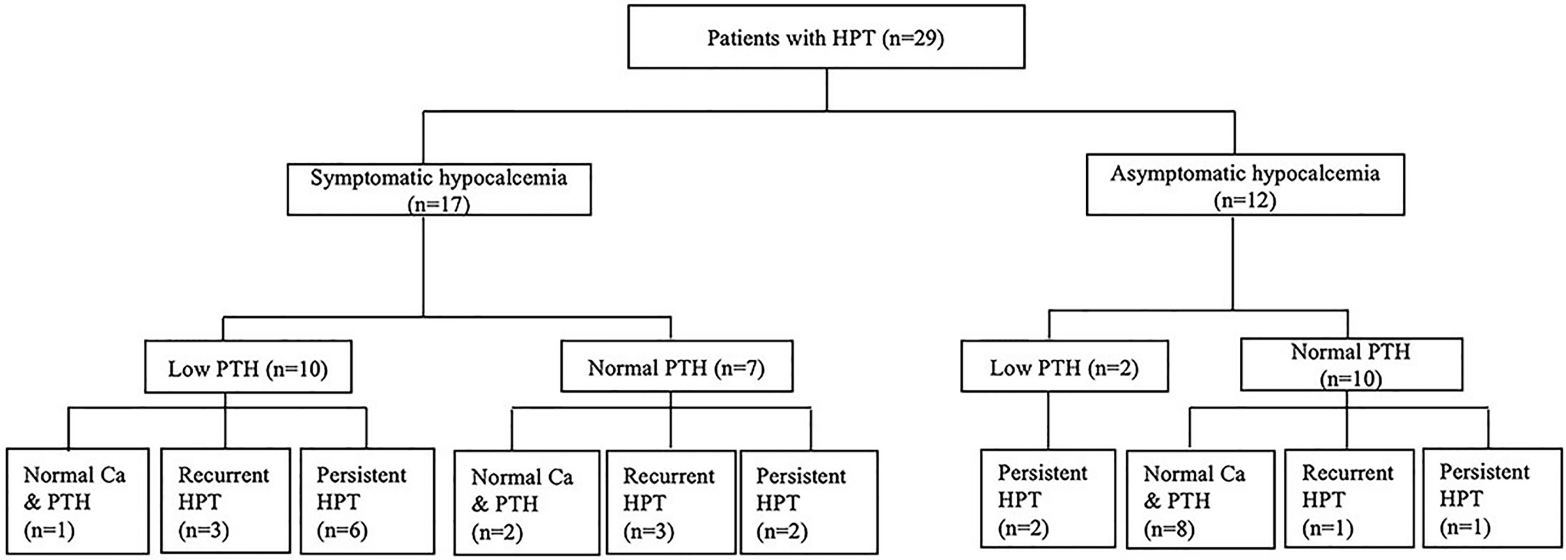
The evolution of parathyroid status in children with 22q11.2 deletion syndrome. Ca, serum calcium level; HPT, hypoparathyroidism; PTH, parathyroid hormone.

In this study, 29 patients with hypoparathyroidism who had been followed up for more than one year were enrolled for further analysis of the natural course of their parathyroid status (**Figure 2**). The mean duration of follow-up was 10.7 ± 8.9 years, and their age at the end of the study was 12.0 ± 9.7 years. At enrollment, 17 patients had symptomatic hypocalcemia as the initial presentation; the other 12 patients were asymptomatic.

Among the 12 patients who were asymptomatic at enrollment, ten had normal iPTH levels. One of these patients remained overt hypoparathyroidism over two years of follow-up. In another case, remission occurred in two months, but he developed overt hypoparathyroidism again 13 years later. The other eight patients had normal calcium and iPTH levels within six months, which remained as such over the ensuing 4.5 ± 2.5 years. The remaining two patients with low iPTH levels at enrollment remained overt hypoparathyroidism over one to two years of follow-up.

Of the 17 patients who had symptomatic hypocalcemia at enrollment, seven had normal iPTH levels. Two of these patients remained overt hypoparathyroidism over two and four years of follow-up. Three other patients had normal calcium and iPTH levels within two years after enrollment but developed overt hypoparathyroidism after nine years, twelve years and thirteen years, remaining so until the end of the study. The other two patients had normal calcium and iPTH levels over one and 16 years of follow-up. Of the remaining ten patients with low iPTH levels at enrollment, overt hypoparathyroidism remained in six cases over 15.7 ± 7.9 years of follow-up. Another patient had normal calcium and iPTH levels within two years after enrollment and remained so over the next 13 years. The other three patients had normal calcium and iPTH levels at one month, three years and five years after enrollment and remained so for one to eight years before overt hypoparathyroidism recurred.

In this study, 18 of 29 patients with hypoparathyroidism who had long-term follow-up still had overt hypoparathyroidism at the end of study. The serum iPTH levels of these 18 patients were 13.3 ± 10.0 pg/mL while the serum iPTH levels of the other 11 patients were 30.4 ± 13.6 pg/mL. The difference was statistically significant (*P*<0.05). Fourteen of 17 patients with symptomatic hypocalcemia at enrollment remained overt hypoparathyroidism at the end of study, as did four of 12 patients with asymptomatic hypocalcemia at enrollment. The difference was also statistically significant (*P*<0.01). In this study, 11 out of 18 patients with overt hypoparathyroidism at the end of study had persistent hypoparathyroidism throughout the course of follow-up while the other seven had a history of recurrent hypoparathyroidism. Two of the latter had overt hypoparathyroidism recurrent at the age of three and six. The other five patients had overt hypoparathyroidism recurrent between 11 years and 14 years of age.

The serum iPTH levels of the 11 patients with persistent hypoparathyroidism were 12.3 ± 10.1 pg/mL while the serum iPTH levels of the other 18 patients with a history of transient hypoparathyroidism were 24.3 ± 14.4 pg/mL. The difference was statistically significant (*P*<0.05). On the other hand, there was no statistically significant difference in the age of onset (*P*=0.387) or in the presence of previous history of symptomatic hypocalcemia (*P*=0.23) between patients with persistent hypoparathyroidism and those with a history of transient hypoparathyroidism.

### Thyroid Disorders

Thyroid disorders were identified in 4 out of 84 evaluated patients with 22q11.2DS (4.8%). Two of them were noted to have right neck swelling at the ages of 24 years and 30 years. However, anti-thyroid antibodies were not detected. Although both of them were euthyroid, thyroid sonography revealed hypoplasia of the left lobe in one and left hemiagenesis of the thyroid gland in the other. Their goiter size decreased after thyroid hormone treatment. The other girl was confirmed to have permanent congenital hypothyroidism detected by neonatal screening, with a blood-spot TSH level of 154 mIU/L, and her thyroid status was fully re-evaluated after thyroid hormone treatment was phased out at the age of three. Her thyroid function test showed FT4 0.561 ng/dL and TSH 279 mIU/L. At that time, the perchlorate discharge test revealed that a radioactive iodine discharge of 64% of the basal uptake measured at two hours after sodium perchlorate administration, suggesting the presence of an iodine organification defect.

Another girl was noted to have goiter with tachycardia at the age of ten. Laboratory tests showed FT4 2.89 ng/dL, TSH 0.02 mIU/L, anti-thyroperoxidase antibody >2000 IU/mL, anti-thyroglobulin antibody >10,000 IU/mL and thyrotropin-binding inhibitory immunoglobulin (TBII) 13.3% (normal <10%), which rose to 56% during follow-up. At that time, her absolute lymphocyte count was 1572 cells per microliter, which was within normal limit. She was treated with carbimazole over the following three years and still under antithyroid therapy at the end of the study.

### Growth Disorders

Twenty-five of 138 patients with 22q11.2DS (18%) were born SGA. Records of height and weight at the last visit were available for 126 patients. Fifty of them (40%) continued to have short stature at the last visit. Four patients with short stature had undergone either insulin hypoglycemic tests or clonidine tests, and all of them had normal growth hormone responses. For further study, their data were divided into four groups according to age. The height standard deviation score (HtSDS) was -2.14 ± 2.56 in 27 patients at an age younger than three, -2.05 ± 1.53 in 41 patients aged between three years and ten years, -1.33 ± 1.07 in 24 patients aged between ten years and eighteen years and -1.19 ± 1.03 in 34 patients at the age older than eighteen (*P*<0.05). Serial follow-up of auxological data in total 33 patients also showed their HtSDS increased gradually with age (odds ratio (OR) 0.06; 95% confidence interval (CI) 0.01-0.11; *P*<0.05). All the results suggested that catch-up growth was present in these patients.

We performed univariate and multivariate logistic regression analysis to identify risk factors for short stature in the 126 patients. As shown in **Table 2**, age, airway anomalies and feeding difficulties due to dysmotility in pharyngoesophageal area were significant risk factors for short stature in univariate analysis. Age had a negative effect on short stature (OR 0.91; 95% CI 0.86-0.96; *P*<0.001). Both airway anomalies (OR 3.80; 95% CI 1.61-8.97; *P*<0.01) and feeding difficulties (OR 4.17; 95% CI 1.35-12.88; *P*<0.05) showed a positive effect on short stature. Although BMI SDS in patients with short stature was significantly lower than those with normal stature (-0.78 ± 1.99 vs. -0.01 ± 2.20, *P*<0.05), BMI SDS only played a marginal effect on short stature in univariate model (OR 0.83; 95% CI 0.69-1.00; *P*=0.0526). After adjusting other risk factors, age (OR 0.91; 95% CI 0.86-0.96; P<0.001) and airway anomalies (OR 2.75; 95% CI 1.04-7.31; P<0.05) remained as significant risk factors for short stature in multivariate model.

**Table 2:** Univariate and multivariate logistic regression analysis of risk factors for short stature.

	Univariate model		Multivariate model	
	OR (95%CI)	*p*-value	OR (95%CI)	*p*-value
Age	0.91 (0.86,0.96)	<0.001	0.91 (0.86,0.96)	<0.001
Congenital heart disease	1.97 (0.76,5.10)	0.16		
Airway anomalies^1^	3.80 (1.61,8.97)	<0.01	2.75 (1.04,7.31)	0.04
Gastrointestinal disease^2^	4.17 (1.35,12.88)	0.01	3.28 (0.90,11.92)	0.07
Cleft palate	2.07 (0.53,8.14)	0.30		
T Lymphopenia^3^	1.18 (0.54,2.59)	0.69		
Recurrent infection	1.85 (0.82,4.16)	0.14		
Small for gestational age	1.82 (0.80,4.18)	0.16		
Hypoparathyroidism	0.88 (0.38,2.06)	0.77		
BMI SDS	0.83 (0.69,1.00)	0.05	0.87 (0.70,1.09)	0.22

^1^subglottic stenosis, tracheomalacia, bronchomalacia and laryngomalacia, ^2^dysmotility in the pharyngoesophageal area with tube feeding, ^3^T cell counts <1500 cells/mm^3^ while younger than 3 years old and <1000 cells/mm^3^ in others. BMI, body mass index; SDS, standard deviation score; OR, odds ratio; CI, confidence interval.

There were 19 patients (14 males and 5 females) for whom both adult height and target height data were available for analysis. Their adult height SDS was -1.15 ± 0.90, and their target height SDS was -0.08 ± 0.65. There was a statistically significant correlation between adult height and target height in these patients (r=0.89, *P*<0.001). However, their adult height SDS was significantly lower than their target height SDS (*P*<0.001).

## Discussion

Hypocalcemia is a common clinical feature in 22q11.2DS. Depending on the sensitivity of recognition and selection criteria, the prevalence of hypocalcemia is estimated at 32% to 80% (1, 4–6). In this study, hypocalcemia was detected in 57 of 135 patients (42%) with confirmed 22q11.2DS, comparable to previous reports (1, 4–6). It is proposed that hypocalcemia in children with 22q11.2DS is invariably due to hypoparathyroidism. However, our results do not agree with this notion, as in a significant proportion of patients with 22q11.2DS in this study, hypocalcemia was related to underlying systemic diseases rather than to hypoparathyroidism per se. Congenital cardiac disease is one of the cardinal features of 22q11.2DS. Patients with severe cardiac defects may have cardiac failure, with poor feeding, respiratory distress, acute renal failure and diuretic use in early life. These systemic problems and their treatment may interfere with calcium homeostasis, such as magnesium depletion, resulting in hypocalcemia in these patients. This is the main reason for the majority of cases with hypocalcemia related to underlying systemic diseases in the study.

Among the other 30 patients with hypocalcemia due to hypoparathyroidism, hypocalcemia was detected in the neonatal period in 18 (60%) and during infancy in eight (27%). Thus, 87% of the patients with 22q11.2DS and hypoparathyroidism experienced their first hypocalcemic event before the age of one, as previously reported (3, 15). Active transport of calcium from mother to fetus is abruptly interrupted at birth, and rapid skeletal growth occurs during the first year of life. Our data suggest that the additional calcium requirement during early infancy may place stress on the reduced parathyroid reserve of these patients, leading to hypocalcemia, especially in individuals who are ill (3).

In this study, more than half of patients with 22q11.2DS and hypoparathyroidism showed serum iPTH levels within the normal range at random evaluation (**Figure 1**), which is similar to previous reports (15, 16). Therefore, random checks of serum iPTH levels alone cannot be used as a sensitive tool for the detection of hypoparathyroidism in patients with 22q11.2DS. Our results also suggest that some degree of parathyroid hormone production is preserved in most patients with 22q11.2DS. However, seven of the 13 patients with 22q11.2DS and hypoparathyroidism shown in **Table 1** had normal iPTH levels of 13–38.9 pg/mL during episodes of symptomatic hypocalcemia, but they were unable to produce more PTH to fully correct their hypocalcemia. Such a finding supports the concept that reduced parathyroid hormone secretory reserve is present in many patients with 22q11.2DS (3, 15, 17). Among the 13 patients shown in **Table 1**, eleven underwent surgery or had concomitant acute illness during episodes of symptomatic hypocalcemia as previously reported (18–20), another patient had symptomatic hypocalcemia after vigorous exercise, and the other patient experienced symptomatic hypocalcemia in adolescence when rapid growth and high bone accretion occur. Our data confirm the idea that the reduced PTH secretory reserve in patients with 22q11.2DS results in a predisposition toward hypocalcemia in the presence of stress or increased calcium demand (16, 18).

Overall, our result demonstrated that patients with low serum iPTH levels at diagnosis or patients with history of symptomatic hypocalcemia more often had overt hypoparathyroidism at the end of the study. Such findings suggest that patients with severe hypoparathyroidism at diagnosis tend to have overt hypoparathyroidism during follow-up. Except for 11 patients with persistent hypoparathyroidism, hypocalcemia in the other 18 patients was transient, with or without recurrence. Both an increase in daily dietary calcium intake and sufficient PTH production by parathyroid glands may allow these patients to achieve normal calcium homeostasis under basal conditions (3, 21), and disodium edetate (EDTA) infusion has been used to unmask latent hypoparathyroidism in these patients (21, 22). We did not arrange for EDTA infusion on our patients. However, the fact that recurrence of hypocalcemia was noted in seven of 18 patients with transient hypocalcemia and that five of them experienced recurrence in adolescence supports this notion. Similar observations have also been reported by Cuneo et al. (23). In this study, we evaluate several factors such as sex, age of onset, serum iPTH level and the presence of history of symptomatic hypocalcemia between patients with persistent hypoparathyroidism and patients with transient hypoparathyroidism to find the risk factors of persistent hypoparathyroidism. We found that only low serum iPTH level was significant as the risk factor for persistent hypoparathyroidism.

Although early infancy and adolescence is the period of time during which hypocalcemia is most likely to be present in patients with 22q11.2DS, hypocalcemia can occur at any age in these patients. Therefore, it is prudent to evaluate the parathyroid function of these patients once every 3–4 months during infancy and then annually during childhood as recommended by Kapadia et al. (15). Our data also confirm the validity of practical guidelines recommended by Bassett et al. (24).

In this series, 3 of 84 patients with 22q11.2DS were confirmed to have permanent primary congenital hypothyroidism. Hence, congenital hypothyroidism is more frequent in those with 22q11.2DS than in the general population in Taiwan (25). Two of them exhibited thyroid developmental abnormalities, one with hypoplasia of the left lobe and the other with left thyroid hemiagenesis. Such findings confirm previous reports that 22q11.2DS is associated with thyroid hypoplasia and dysgenesis (24, 26, 27). Thyroid hemiagenesis is a rare congenital disorder of the thyroid with an estimated prevalence of 0.05%-0.5% (28–30). Because compensatory growth of the residual tissue permits adequate thyroid hormone production in most patients with thyroid hemiagenesis, they are frequently clinically euthyroid (30–32). On the other hand, chronic endogenous TSH overstimulation may partially account for the presence of a variety of pathological conditions overlapping thyroid hemiagenesis in the literature (30–32). It is not surprising, therefore, to observe that both our patients had compensatory hypertrophy of the residual right lobe in their third decade of life. The other patient with 22q11.2DS developed dyshormongenesis due to organification defects. To our knowledge, this is the first related case report in the literature. We did not find any pathogenic mutation in the thyroperoxidase, *DUOX2*, *DUOXA2* and thyroglobulin genes of this patient (data not shown). Further studies are required to clarify the relationship between dyshormongenesis of the thyroid and 22q11.2DS.

Graves’ disease is reported to be an uncommon but significant manifestation of 22q11.2DS (33, 34). Although the mechanism remains unknown, it has been proposed that 22q11.2DS is associated with a predisposition to autoimmune thyroid disease (33–35). Our results are consistent with such a notion.

The prevalence of short stature in patients with 22q11.2DS was 40% in this study, which was somewhat higher than that reported in previous studies (1, 8, 16). The serial follow-up of auxological data in our patients revealed the tendency of catch-up growth in patients with 22q11.2DS. The tendency to have catch-up growth can explain younger age as the most important risk factor in our multivariate logistic regression model. This growth pattern may be partially explained by constitutional growth delay or impaired nutritional status due to multiple congenital anomalies after birth in these patients (1, 8, 10), which improved with age. Our results also showed airway anomalies as another risk factor for short stature in multivariate logistic regression model. Nearly all (28 of 30) of our patients with airway anomalies also had congenital heart disease and underwent major operation during the period of early infancy. The presence of airway anomalies, which implied the most severe form of congenital heart disease in patients with 22q11.2DS, may affect their hospitalization course (36) and growth potential. Such a finding has also been reported by Levy-Shraga et al. (7). Final adult height of our patients with 22q11.2DS had a significant correlation with their target height. However, their adult height was shorter than their target height or adult height of normal population, which was in agreement with previously reports (7, 10). It has been reported the prevalence of growth hormone deficiency is estimated to be 4% in patients with 22q11.2DS and supplement of growth hormone may improve their growth (9). In our limited experience, we did not find patients with growth hormone deficiency.

In conclusion, hypoparathyroidism is a common endocrine disorder in patients with 22q11.2 DS. However, our data demonstrated that hypocalcemia in patients with 22q11.2 DS is not always due to hypoparathyroidism per se and careful differential diagnosis to clarify the causes is advised. Patients with 22q11.2DS have a long-term risk of overt hypoparathyroidism at any age, even in those with some parathyroid hormone reserve. Therefore, it is prudent to conduct a regular survey of parathyroid function at diagnosis and follow-up, especially during infancy, adolescence or in the presence of stress, such as surgery or acute illness. Thyroid disorders were present in 4.8% of patients with 22q11.2DS in this study so thyroid function testing at diagnosis or in the presence of symptoms is justified. Short stature is another concern in these patients, especially in those with severe cardiovascular illness associated with airway anomalies. There is a tendency of catch-up growth but adult height is still compromised. Regular monitoring of endocrine function and growth is indicated in order to supply appropriate treatment in time.

## Data Availability Statement

The original contributions presented in the study are included in the article/supplementary material. Further inquiries can be directed to the corresponding author.

## Ethics Statement

The studies involving human participants were reviewed and approved by Research Ethics Committee B National Taiwan University Hospital 7, Chung Shan South Road, Taipei, Taiwan 100, R.O.C Phone: 886-2-23123456 Fax: 886-2-23951950. Written informed consent from the participants’ legal guardian/next of kin was not required to participate in this study in accordance with the national legislation and the institutional requirements.

## Author Contributions

H-YL, W-YT, and C-TL analyzed the data and wrote the article. C-TL had primary responsibility for final content. Y-CT and S-YL provided primary patient data. N-CL, Y-HC, and W-LH performed the genetic tests and provided primary patient data. C-TL conceived and designed research. All authors contributed to the article and approved the submitted version.

## Conflict of Interest

The authors declare that the research was conducted in the absence of any commercial or financial relationships that could be construed as a potential conflict of interest.

## Publisher’s Note

All claims expressed in this article are solely those of the authors and do not necessarily represent those of their affiliated organizations, or those of the publisher, the editors and the reviewers. Any product that may be evaluated in this article, or claim that may be made by its manufacturer, is not guaranteed or endorsed by the publisher.
